# Subgroup analysis of the AFTER I-O study: a retrospective study on the efficacy and safety of subsequent molecular targeted therapy after immune-oncology therapy in Japanese patients with metastatic renal cell carcinoma

**DOI:** 10.1093/jjco/hyab114

**Published:** 2021-08-04

**Authors:** Yoshihiko Tomita, Go Kimura, Satoshi Fukasawa, Kazuyuki Numakura, Yutaka Sugiyama, Kazutoshi Yamana, Sei Naito, Hirokazu Kaneko, Yohei Tajima, Mototsugu Oya

**Affiliations:** Department of Urology, Molecular Oncology, Graduate School of Medicine and Dental Sciences, Niigata University, Niigata, Japan; Department of Urology, Nippon Medical School Hospital, Tokyo, Japan; Prostate Center and Division of Urology, Chiba Cancer Center, Chiba, Japan; Department of Urology, Akita University Graduate School of Medicine, Akita, Japan; Department of Urology, Graduate School of Medical Sciences, Kumamoto University, Kumamoto, Japan; Department of Urology, Molecular Oncology, Graduate School of Medicine and Dental Sciences, Niigata University, Niigata, Japan; Department of Urology, Yamagata University Faculty of Medicine, Yamagata, Japan; Bristol-Myers Squibb, Tokyo, Japan; Ono Pharmaceutical Co., Ltd., Osaka, Japan; Department of Urology, Keio University School of Medicine, Tokyo, Japan

**Keywords:** renal cell carcinoma, molecular targeted therapy, nivolumab, ipilimumab

## Abstract

**Background:**

We performed subgroup analyses of the AFTER I-O study to clarify the association of time-to-treatment failure (TTF) and discontinuation reason of prior immune-oncology (I-O) therapy, and molecular targeted therapy (TT) regimen with the outcomes of TT after I-O.

**Methods:**

The data of Japanese metastatic renal cell carcinoma patients treated with TT after nivolumab (NIVO) (CheckMate 025) or NIVO + ipilimumab (IPI) (CheckMate 214) were retrospectively analyzed. The objective response rates (ORRs), progression-free survival (PFS) and overall survival (OS) of TT after I-O were analyzed by subgroups: TTF (<6 or ≥6 months) and discontinuation reason of prior I-O (progression or adverse events), and TT regimen (sunitinib or axitinib). We also analyzed PFS2 of prior I-O and OS from first-line therapy.

**Results:**

The ORR and median PFS of TT after NIVO and NIVO+IPI among the subgroups was 17–36% and 20–44%, and 7.1–11.6 months and 16.3-not reached (NR), respectively. The median OS of TT after NIVO was longer in patients with longer TTF of NIVO and treated with axitinib. Conversely, median OS of TT after NIVO+IPI was similar among subgroups. The median PFS2 of NIVO and NIVO+IPI was 36.7 and 32.0 months, respectively. The median OS from first-line therapy was 70.5 months for patients treated with NIVO and NR with NIVO+IPI. The safety profile of each TT after each I-O was similar to previous reports.

**Conclusions:**

The efficacy of TT after NIVO or NIVO+IPI was favorable regardless of the TTF and discontinuation reason of prior I-O, and TT regimen.

## Teaser

Efficacy of molecular targeted therapy after immune-oncology therapy was favorable without new safety signals, regardless of time to treatment failure and reason for discontinuation of prior I-O, and TT regimen.

## Introduction

The prognosis of patients with metastatic renal cell carcinoma (mRCC) has dramatically improved in the immuno-oncology (I-O) era than in the cytokine (1) and molecular targeted therapy (TT) eras (2–4). Many treatment options have been approved as first-line mRCC therapy, such as I-O combination therapies including nivolumab and ipilimumab (NIVO+IPI), pembrolizumab and axitinib, avelumab and axitinib, and classical vascular endothelial growth factor receptor tyrosine kinase inhibitor (VEGFR-TKI) monotherapies. Moreover, as for second- or later-line therapy, nivolumab (NIVO), cabozantinib, axitinib and everolimus have been approved. Among these treatment options, the design of sequential therapy is an important factor for the prognosis of mRCC patients; however, the lack of diversity of mechanisms of action for treating mRCC causes difficulties regarding switching the mechanism of action between treatment lines.

Most of the outcomes of subsequent therapy observed in pivotal clinical trials are insufficient, and long-term follow-ups and observational studies of subsequent therapy in real-world settings are needed for decision-making regarding treatment strategies. Consistent with previous reports on the outcomes of subsequent therapy after NIVO or immuno-oncology (I-O) combination therapy (5–21), we have also reported favorable anti-tumor activity of TT after the discontinuation of NIVO or NIVO+IPI in mRCC patients in Japanese real-world settings in the ‘AFTER I-O study’ (22).

Iacovelli *et al.* (16) only reported the correlation between the outcomes of I-O treatment and efficacy of subsequent therapy. Furthermore, certain reports are available regarding the efficacy of specific TT after I-O regimens (10,13,15,16,18–20).

In this study, we analyzed the outcome of the AFTER I-O study by subgroups: length of time-to-treatment failure (TTF), reason for discontinuation of NIVO or NIVO+IPI, and first TT regimen after NIVO or NIVO+IPI (sunitinib or axitinib). Moreover, we made additional analyses on progression-free survival 2 (PFS2) after NIVO and NIVO+IPI and overall survival (OS) from first-line therapy to clarify the long-term benefit of NIVO and NIVO+IPI.

## Patients and methods

The ‘AFTER I-O study’ was a multicenter, retrospective, observational study conducted in Japan. This study analyzed patients that participated in the CheckMate 025 or CheckMate 214 trials and were treated with TT as a subsequent therapy before 31 March 2019, and after the discontinuation of NIVO or NIVO+IPI. The primary endpoints were the objective response rates (ORRs) to the first TT after discontinuation of NIVO or NIVO+IPI. The secondary endpoints included the efficacy of TT after NIVO or NIVO+IPI, such as PFS, OS and safety. Additionally, we analyzed PFS2 after NIVO and NIVO+IPI and OS from first-line therapy. PFS was defined as the time from the first TT dose after NIVO or NIVO+IPI to disease progression (PD) or death. PFS2 was defined as the time from the first dose of NIVO or NIVO+IPI to PD or death during subsequent therapy with the first TT after NIVO or NIVO+IPI. The OS of TT was defined as the time from the first TT dose after NIVO or NIVO+IPI to death and the OS of first-line therapy as the time from first-line therapy to death.

The AFTER I-O study was approved by the Ethics Committee of Niigata University (Approval Number: 2018–0416, Date: 10 April 2019) and other independent institutional review boards and was conducted according to the Declaration of Helsinki and Ethical Guidelines for Medical and Health Research Involving Human Subjects. This study is registered in the University hospital Medical Information Network under number UMIN000036063. This retrospective study used medical records for analysis, and thus, informed consent from patients was not required. This paper does not disclose any personally identifiable information of any of the participants in any form. Hence, consent for publication is not applicable.

### Statistical analyses

OS, PFS and PFS2 were estimated by the Kaplan–Meier method, and the 95% confidence intervals for each subgroup were determined using hierarchical Bayesian survival analysis and Cox’s proportional hazards model. SAS (SAS Institute Japan Ltd., version 9.4) was used for all analyses. The efficacy of the first TT after discontinuation of NIVO or NIVO+IPI was analyzed in the next subgroups: TTF of NIVO or NIVO+IPI, cutoff at 6 months; reason for discontinuation of NIVO or NIVO+IPI, PD or adverse event; regimen of TT after discontinuation of NIVO or NIVO+IPI, sunitinib or axitinib, and safety were analyzed in subgroups of TT regimens, sunitinib or axitinib. Statistical differences were not tested between any subgroups due to the small size of the study.

**Table 1:** Patient characteristics at the start of the first targeted therapy (TT) after the discontinuation of nivolumab or nivolumab and ipilimumab combination therapy

		CheckMate 025					
		All *N* = 26		Sunitinib *N* = 8		Axitinib *N* = 14	
Sex, *n* (%)	Male	17	(65)	5	(63)	8	(57)
	Female	9	(35)	3	(38)	6	(43)
Age, years	Median (range)	69.0	(40–83)	65.0	(52–79)	70.0	(40–83)
Regimens before ICI, *n* (%)	1	14	(54)	4	(50)	8	(57)
	2	8	(31)	3	(38)	5	(36)
	3	4	(15)	1	(13)	1	(7)
TTF of ICI, months	Median (range)	9.4	(0.5–59.4)	7.2	(0.7–25.5)	10.1	(0.5–59.4)
Reason for ICI discontinuation, *n* (%)	Progression	20	(77)	6	(75)	10	(71)
	Adverse events	6	(23)	2	(25)	4	(29)
Surgery after ICI discontinuation, *n* (%)	Yes	1	(4)	0	(0)	1	(7)
	No	25	(96)	8	(100)	13	(93)
ECOG PS, *n* (%)	0	20	(77)	7	(88)	11	(79)
	1	4	(15)	1	(13)	1	(7)
	≥2	1	(4)	0	(0)	1	(7)
	Unknown	1	(4)	0	(0)	1	(7)
MSKCC risk classification at 1st subsequent TT after ICI, *n* (%)	Favorable	6	(23)	3	(38)	2	(14)
	Intermediate	14	(54)	5	(63)	7	(50)
	Poor	4	(15)	0	(0)	3	(21)
	Unknown	2	(8)	0	(0)	2	(14)
Primary tumor	Yes	5	(19)	2	(25)	2	(14)
Metastatic site	Lung	19	(73)	4	(50)	11	(79)
	Bone	6	(23)	2	(25)	4	(29)
	Brain	2	(8)	1	(13)	0	(0)
	Liver	7	(27)	3	(38)	4	(29)
	Lymph node	9	(35)	4	(50)	4	(29)
CRP ≥ upper limit of facility normal, *n* (%)	Yes	18	(69)	5	(63)	10	(71)
		CheckMate 214 (all risks)					
		All *N* = 19		Sunitinib *N* = 6		Axitinib *N* = 9	
Sex, *n* (%)	Male	17	(90)	6	(100)	7	(78)
	Female	2	(11)	0	(0)	2	(22)
Age, years	Median (range)	70.0	(45–82)	60.0	(46–73)	77.0	(45–82)
TTF of ICI, months	Median (range)	6.2	(0.0–27.6)	5.5	(0.0–27.6)	4.7	(1.4–25.7)
Reason for ICI discontinuation, *n* (%)	Progression	13	(68)	4	(67)	6	(67)
	Adverse events	6	(32)	2	(33)	3	(33)
Surgery after ICI discontinuation, *n* (%)	Yes	3	(16)	0	(0)	1	(11)
	No	16	(84)	6	(100)	8	(89)
ECOG PS, *n* (%)	0	10	(53)	4	(67)	3	(33)
	1	5	(26)	1	(17)	3	(33)
	≥2	3	(16)	1	(16.7)	2	(22)
	Unknown	1	(5)	0	(0)	1	(11)
IMDC risk classification at 1st subsequent TT after ICI, *n* (%)	Favorable	1	(5)	1	(17)	0	(0)
	Intermediate	14	(74)	4	(67)	7	(78)
	Poor	3	(16)	1	(17)	1	(11)
	Unknown	1	(5)	0	(0)	1	(11)
Primary tumor	Yes	3	(16)	1	(17)	2	(22)
Metastatic site	Lung	12	(63)	4	(67)	6	(67)
	Bone	7	(37)	2	(33)	2	(22)
	Brain	1	(5)	1	(17)	0	(0)
	Liver	3	(16)	0	(0)	2	(22)
	Lymph node	8	(42)	3	(50)	4	(44)
CRP ≥ upper limit of facility normal, *n* (%)	Yes	13	(68)	4	(67)	6	(67)

## Results

The patient characteristics are summarized in Table 1, and patient characteristics of intermediate/poor risks of CheckMate 214 are summarized in Table S1. A total of 45 mRCC patients from 20 Japanese centers were retrospectively analyzed, including 26 out of 37 Japanese patients treated with NIVO in CheckMate 025 (23,24) and 19 out of 38 Japanese patients (all risks) treated with NIVO+IPI in CheckMate 214 (25). The median follow-up period from the start of the first TT after discontinuation of NIVO or NIVO+IPI to the date of analysis or death was 22.1 months (range: 3.2–65.4 months) for patients from CheckMate 025 and 20.3 months (range: 1.1–39.9 months) for patients from CheckMate 214 (all risks). The median follow-up period from the start of first-line therapy to the date of analysis or death was 70.2 months (range: 21.7–125.6 months) and 42.1 months (range: 2.7–48.4 months) for patients from CheckMate 025 and CheckMate 214 (all risks), respectively.

**Figure 1.**
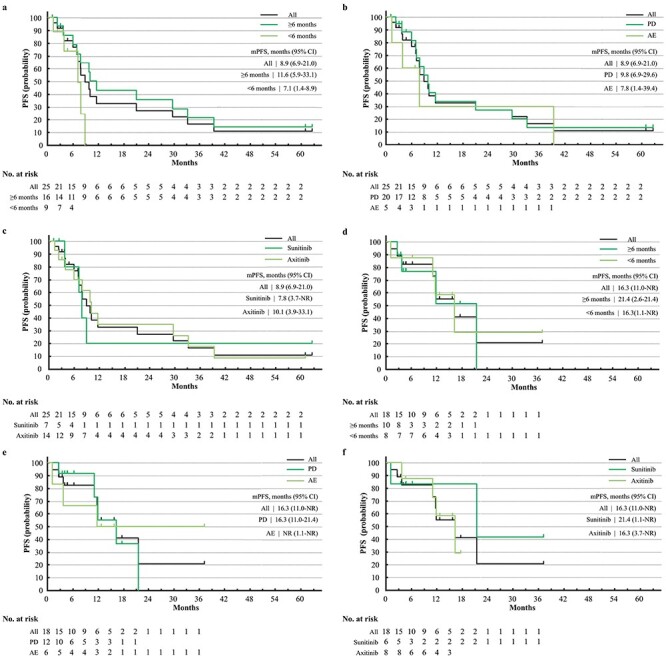
Progression-free survival (PFS) of targeted therapy (TT) after discontinuation of nivolumab (NIVO) or nivolumab and ipilimumab combination therapy (NIVO+IPI). (a) PFS of TT after discontinuation of NIVO, stratified by time-to-treatment failure (TTF) of NIVO, with a cutoff value at 6 months. (b) PFS of targeted therapy after discontinuation of NIVO, stratified by reason for discontinuation of NIVO, disease progression or adverse events. (c) PFS of TT after discontinuation of NIVO, stratified by TT regimens after NIVO, sunitinib or axitinib. (d) PFS of TT after discontinuation of NIVO+IPI, stratified by TTF of NIVO+IPI, with a cutoff value at 6 months, IMDC all risks. (e) PFS of TT after discontinuation of NIVO+IPI, stratified by reason for discontinuation of NIVO+IPI, disease progression or adverse events, IMDC all risks. (f) PFS of TT after discontinuation of NIVO+IPI, stratified by TT regimens after NIVO+IPI, sunitinib or axitinib, IMDC all risks.

**Figure 2.**
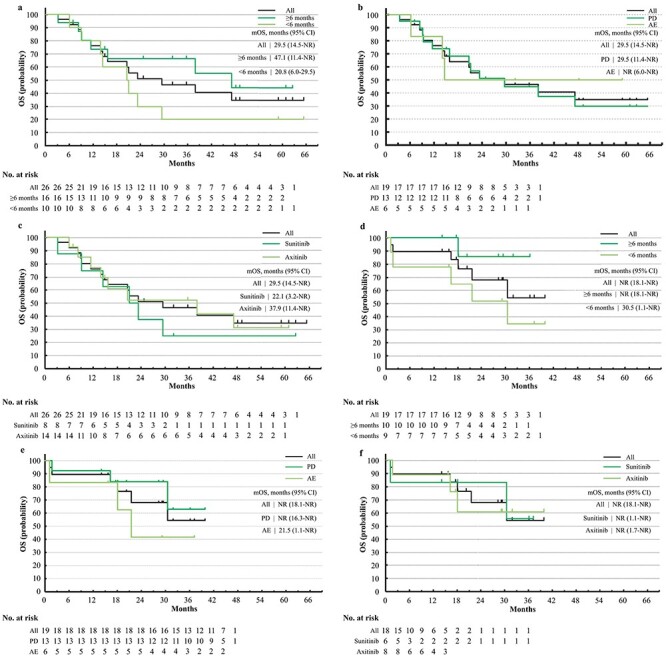
Overall survival (OS) of TT after discontinuation of nivolumab (NIVO) or nivolumab and ipilimumab combination therapy (NIVO+IPI). (a) OS of TT after discontinuation of NIVO, stratified by TTF of NIVO, with a cutoff value at 6 months. (b) OS of TT after discontinuation of NIVO, stratified by reason for discontinuation of NIVO, disease progression or adverse events. (c) OS of TT after discontinuation of NIVO, stratified by TT regimens after NIVO, sunitinib or axitinib. (d) OS of TT after discontinuation of NIVO+IPI, stratified by TTF of NIVO+IPI, with a cutoff value at 6 months, IMDC all risks. (e) OS of TT after discontinuation of NIVO+IPI, stratified by reason for discontinuation of NIVO+IPI, disease progression or adverse events, and IMDC all risks. (f) OS of TT after discontinuation of NIVO+IPI, stratified by TT regimens after NIVO+IPI, sunitinib or axitinib, IMDC all risks.

**Figure 3.**
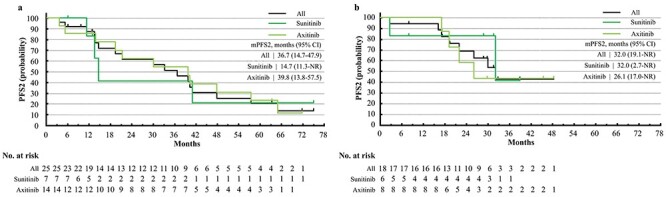
PFS2 of nivolumab (NIVO) or nivolumab and ipilimumab combination therapy (NIVO+IPI). (a) PFS2 of NIVO, stratified by TT regimens after NIVO, sunitinib, or axitinib. (b) PFS2 of NIVO+IPI, stratified by TT regimens after NIVO+IPI, sunitinib, or axitinib, IMDC all risks.

**Figure 4.**
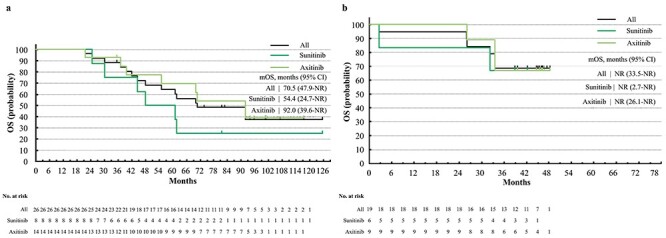
OS from first-line therapy of patients treated with nivolumab (NIVO) or nivolumab and ipilimumab combination therapy (NIVO+IPI). (a) OS from first-line therapy of patients treated with NIVO, stratified by TT regimens after NIVO, sunitinib or axitinib. (b) OS from first-line therapy of patients treated with NIVO+IPI, stratified by TT regimens after NIVO+IPI, sunitinib or axitinib, IMDC all risks.

The ORR and BOR of all the patients and their subgroups are summarized in Table 2 for the patients from CheckMate 025 and in Table 3 for the patients from CheckMate 214 (all risks). Data for IMDC intermediate/poor risks are summarized in Table S2. Kaplan–Meier curves of PFS, PFS2, OS of TT, and OS from first-line therapy are shown in Figs 1–4, respectively. Data for patients from CheckMate 214 and the intermediate/poor risks are shown in Figs S1–S4.

**Table 2:** Overall response rate and BOR of TT after the discontinuation of nivolumab, subgroup in time-to-treatment failure of nivolumab, reason for discontinuation of nivolumab and TT regimens

		All		TTF of NIVO				Reason for discontinuation of NIVO				TT regimen			
				≥6 months		<6 months		PD		AE		Sunitinib		Axitinib	
		*N* = 26		*N* = 16		*N* = 10		*N* = 20		*N* = 6		*N* = 8		*N* = 14	
ORR, *n* (%)		7	(27)	5	(31)	2	(20)	6	(30)	1	(17)	2	(25)	5	(36)
DCR, *n* (%)		23	(88)	15	(94)	8	(80)	19	(95)	4	(67)	7	(88)	12	(86)
BOR, *n* (%)	CR	0	(0)	0	(0)	0	(0)	0	(0)	0	(0)	0	(0)	0	(0)
	PR	7	(27)	5	(31)	2	(20)	6	(30)	1	(17)	2	(25)	5	(36)
	SD	16	(62)	10	(63)	6	(60)	13	(65)	3	(50)	5	(63)	7	(50)
	PD	2	(8)	1	(6)	1	(10)	1	(5)	1	(17)	0	(0)	2	(14)
	NE	1	(4)	0	(0)	1	(10)	0	(0)	1	(17)	1	(13)	0	(0)

**Table 3:** Overall response rate and BOR of TT after the discontinuation of nivolumab and ipilimumab combination therapy (NIVO+IPI), subgroup in time-to-failure of NIVO+IPI, reason for discontinuation of NIVO+IPI, TT regimens, IMDC all risks

		All		TTF of NIVO+IPI				Reason for discontinuation of NIVO+IPI				TT regimen			
				≥6 months		<6 months		PD		AE		Sunitinib		Axitinib	
		*N* = 19		*N* = 10		*N* = 9		*N* = 13		*N* = 6		*N* = 6		*N* = 9	
ORR, *n* (%)		6	(32)	2	(20)	4	(44)	4	(31)	2	(33)	2	(33)	4	(44)
DCR, *n* (%)		16	(84)	8	(80)	8	(89)	11	(85)	5	(83)	6	(100)	7	(78)
BOR, *n* (%)	CR	0	(0)	0	(0)	0	(0)	0	(0)	0	(0)	0	(0)	0	(0)
	PR	6	(32)	2	(20)	4	(44)	4	(31)	2	(33)	2	(33)	4	(44)
	SD	10	(53)	6	(60)	4	(44)	7	(54)	3	(50)	4	(67)	3	(33)
	PD	2	(11)	2	(20)	0	(0)	1	(8)	1	(17)	0	(0)	1	(11)
	NE	1	(5)	0	(0)	1	(11)	1	(8)	0	(0)	0	(0)	1	(11)

**Table 4:** Treatment-related adverse events of TT after nivolumab or nivolumab and ipilimumab combination therapy occurring in >15% of patients stratified by TT regimens after nivolumab or nivolumab and ipilimumab combination therapy, sunitinib or axitinib

	CheckMate 025 + CheckMate 214					
	All *N* = 45		Sunitinib *N* = 14		Axitinib *N* = 23	
	Any grade	Grades 3–4	Any grade	Grades 3–4	Any grade	Grades 3–4
Treatment-related adverse events, *n* (%)	44 (97.8)	23 (51.1)	14 (100.0)	10 (71.4)	22 (95.7)	9 (39.1)
Hypertension	17 (37.8)	4 (8.9)	3 (21.4)	0 (0.0)	11 (47.8)	3 (13.0)
Fatigue	16 (35.6)	1 (2.2)	7 (50.0)	1 (7.1)	6 (26.1)	0 (0.0)
Hoarseness	15 (33.3)	0 (0.0)	1 (7.1)	0 (0.0)	13 (56.5)	0 (0.0)
Anorexia	14 (31.1)	3 (6.7)	5 (35.7)	2 (14.3)	8 (34.8)	1 (4.3)
Platelet count decreased	13 (28.9)	4 (8.9)	8 (57.1)	3 (21.4)	3 (13.0)	0 (0.0)
Proteinuria	13 (28.9)	2 (4.4)	1 (7.1)	0 (0.0)	8 (34.8)	2 (8.7)
Hypothyroidism	13 (28.9)	1 (2.2)	3 (21.4)	1 (7.1)	8 (34.8)	0 (0.0)
Palmar-plantar erythrodysesthesia syndrome	12 (26.7)	1 (2.2)	4 (28.6)	0 (0.0)	7 (30.4)	1 (4.3)
Diarrhea	12 (26.7)	0 (0.0)	2 (14.3)	0 (0.0)	9 (39.1)	0 (0.0)
Anemia	11 (24.4)	1 (2.2)	6 (42.9)	1 (7.1)	3 (13.0)	0 (0.0)
Creatinine increased	8 (17.8)	0 (0.0)	3 (21.4)	0 (0.0)	3 (13.0)	0 (0.0)
Aspartate aminotransferase increased	7 (15.6)	4 (8.9)	3 (21.4)	2 (14.3)	2 (8.7)	1 (4.3)
White blood cell decreased	7 (15.6)	2 (4.4)	6 (42.9)	2 (14.3)	1 (4.3)	0 (0.0)
Lymphocyte count decreased	7 (15.6)	2 (4.4)	5 (35.7)	1 (7.1)	2 (8.7)	1 (4.3)

The ORR of TT after NIVO varied from 17 to 36% (Table 2), while that after NIVO+IPI varied from 20 to 44% (Table 3). The median PFS of TT after NIVO varied from 7.1 to 11.6 months among subgroups (Fig. 1a–c) and from 16.3 months to not reached (NR) for TT after NIVO+IPI (Fig. 1d–f). The median OS of TT after NIVO was longer in patients who had a longer TTF of NIVO (47.1 vs. 20.8 months) and in patients treated with axitinib vs. sunitinib (37.9 vs. 22.1 months). Conversely, the median OS of TT after NIVO+IPI was relatively similar among subgroups (Fig. 2d–f). The median PFS2 of NIVO and NIVO+IPI was 36.7 and 32.0 months, respectively (Fig. 3). The median OS from first-line therapy was 70.5 months for patients treated with NIVO and NR for patients treated with NIVO+IPI (Fig. 4). The median PFS2 and OS from first-line therapy of patients treated with NIVO were longer in patients treated with axitinib after NIVO than in patients treated with sunitinib (39.8 vs. 14.7 months and 92.0 vs. 54.4 months, Figs 3c and 4c).

The safety data are summarized in Table 4. All patients treated with sunitinib after NIVO or NIVO+IPI and almost all patients treated with axitinib experienced treatment-related adverse events, of which grade 3–4 events were more common in patients treated with sunitinib than in those treated with axitinib. Finally, no treatment-related deaths and no new safety signals were reported.

## Discussion

This subgroup analysis of the AFTER I-O study revealed that the efficacy of TT after NIVO or NIVO+IPI was promising, regardless of the TTF of NIVO or NIVO+IPI, reason for discontinuation of I-O therapy, or TT regimen, sunitinib or axitinib.

To date, three studies have reported that patients with short PFS or TTF of first-line VEGFR-TKI had poorer prognoses (4,26,27). In this subgroup analysis, PFS and OS of TT after first-line NIVO+IPI were similar among subgroups of TTF cut-off at 6 months (Figs 1d and 2d). However, the median OS of TT after NIVO+IPI with a short TTF of NIVO+IPI (30.5 months) was longer than the OS of the RECORD-1 (everolimus, median OS: 14.8 months), AXIS (axitinib after subitinib, median OS: 15.2 months) and METEOR (cabozantinib, median OS: 21.4 months) trials (28–30), and real world data of axitinib in Japan reported by Miyake *et al.* (median OS: 27.0 months) (31).

As for TT-TT sequential therapy, many studies have reported patients who discontinued TT due to PD had poorer PFS and OS than those that discontinued due to adverse events (32–35). Such a trend was not present in our current analysis of either TT after NIVO (Table 2, Fig. 1b and e) or NIVO+IPI (Table 3, Fig. 2b and e), indicating the advantage of changing the mechanism of action between treatment lines.

Ishihara *et al.* (18) reported the efficacy of third-line axitinib after second-line NIVO (ORR: 29.4%, median PFS: 12.8 months, median OS: NR), and Yasuoka *et al.* (20) also reported the efficacy of third- or fourth-line axitinib after NIVO (ORR: 56.3%, median PFS: 7.9 months, median OS: NR). This analysis is the third that reports favorable efficacy of axitinib after NIVO. There are no other available reports regarding the efficacy of sunitinib or axitinib after NIVO+IPI.

Although the median OS of sunitinib was shorter than that of axitinib after NIVO (22.1 vs. 37.9 months, Fig. 2c), the median OS of sunitinib after NIVO was comparable or longer than that in the pivotal studies, such as RECORD-1, AXIS and METEOR (28–30). The median PFS of axitinib after NIVO (10.1 months) and NIVO+IPI (16.3 months) was similar to the Japanese subgroup analysis of the AXIS study which included first-line cytokine and TT therapy (median PFS: 12.1 months) (36).

The PFS2 and OS from the first-line therapies of the RECORD-3, SWICH, SWICH II and CROSS-J-RCC trials were previously reported for TT sequential therapy (37–40). The JAVELIN Renal 101 study was the only previous report of PFS2 in the I-O era (41). In the present study, the median PFS2 (36.7 and 32.0 months for patients from CheckMate 025 and CheckMate 214, respectively) and the median OS from first-line therapy (70.5 months for patients from CheckMate 025) were also longer than the previous reports on TT sequential therapy (median PFS2: 8.6–27.8 months; median OS from first-line therapy: 22.4–38.9 months). The median PFS2 was longer for patients from CheckMate 025 than CheckMate 214, probably because the patients from CheckMate 214 with long PFS after NIVO+IPI were not treated with subsequent TT by the time of analysis. The median OS from first-line therapy of patients treated with axitinib after NIVO exceeded 7 years, which is a remarkable data. In the I-O era, systemic therapy may result in OS of more than 7 years, which will be encouraging for the patients of mRCC.

The safety profile of sunitinib or axitinib after NIVO and NIVO+IPI was consistent with previous reports and without new safety signals.

The AFTER I-O study had some limitations: first the nature of the retrospective study, and second, the small sample size; as each subgroup was too small, comparisons between subgroups should be performed with caution.

In conclusion, the efficacy of TT after NIVO or NIVO+IPI was favorable independent of the TTF and the reason for discontinuation of NIVO or NIVO+IPI, and TT regimen.

## Data availability statement

The datasets generated during and/or analyzed during the current study are available from the corresponding author on reasonable request.

## Author contributions

Tomita, Kaneko, and Tajima have full access to all the data in the study and take responsibility for the integrity of the data and the accuracy of the data analysis.

Study concept and design: Tomita, Kaneko, and Tajima.

Provision of study materials or patients: Tomita, Kimura, Fukasawa, Numakura, Sugiyama, Yamana, Naito, and Oya.

Collection and assembly of data: Tomita, Kaneko, and Tajima.

Data analysis and interpretation: All authors.

Drafting of the manuscript: Tomita, Kaneko, and Tajima.

Critical revision of the manuscript for important intellectual content: All authors.

Final approval of manuscript: All authors.

## Supplementary Material

Supplemetary_Tables_and_Figures_hyab114

## Prior presentation

Go Kimura has received honoraria from Bayer, Bristol-Myers Squibb, Novartis, Ono Pharmaceutical Co., Ltd. and Pfizer.

Satoshi Fukasawa, Kazuyuki Numakura, Yutaka Sugiyama, Kazutoshi Yamana and Sei Naito do not have any conflicts of interest to disclose.

Hirokazu Kaneko is an employee of Bristol-Myers Squibb.

Yohei Tajima is an employee of Ono Pharmaceutical Co., Ltd.

Mototsugu Oya has received honoraria from Bayer, Bristol-Myers Squibb, Novartis, Ono Pharmaceutical Co., Ltd. and Pfizer.

## Notes

Presented at the 58th Annual Meeting of the Japan Society of Clinical Oncology, October 22–25, 2020, at Kyoto, Japan.
