# Erratum to: Subgroup analysis of the AFTER I-O study: a retrospective study on the efficacy and safety of subsequent molecular targeted therapy after immune-oncology therapy in Japanese patients with metastatic renal cell carcinoma

**DOI:** 10.1093/jjco/hyab161

**Published:** 2021-09-29

**Authors:** Yoshihiko Tomita, Go Kimura, Satoshi Fukasawa, Kazuyuki Numakura, Yutaka Sugiyama, Kazutoshi Yamana, Sei Naito, Hirokazu Kaneko, Yohei Tajima, Mototsugu Oya

**Affiliations:** 1 Department of Urology, Molecular Oncology, Graduate School of Medicine and Dental Sciences, Niigata University, Niigata, Japan; 2 Department of Urology, Nippon Medical School Hospital, Tokyo, Japan; 3 Prostate Center and Division of Urology, Chiba Cancer Center, Chiba, Japan; 4 Department of Urology, Akita University Graduate School of Medicine, Akita, Japan; 5 Department of Urology, Graduate School of Medical Sciences, Kumamoto University, Kumamoto, Japan; 6 Department of Urology, Yamagata University Faculty of Medicine, Yamagata, Japan; 7 Bristol-Myers Squibb, Tokyo, Japan; 8 Ono Pharmaceutical Co., Ltd., Osaka, Japan; 9 Department of Urology, Keio University School of Medicine, Tokyo, Japan

In the originally published article there were errors in the legends to figures 3 and 4.

Figure 3 should read: “Figure 3. PFS2 of nivolumab (NIVO) or nivolumab and ipilimumab combination therapy (NIVO+IPI). (a) PFS2 of NIVO, stratified by TT regimens after NIVO, sunitinib, or axitinib. (b) PFS2 of NIVO+IPI, stratified by TT regimens after NIVO+IPI, sunitinib, or axitinib, IMDC all risks.” instead of: “Figure 3. OS from first-line therapy of patients treated with nivolumab (NIVO) or nivolumab and ipilimumab combination therapy (NIVO+IPI). (a) OS from first-linetherapy of patients treated with NIVO, stratified by TT regimens after NIVO, sunitinib or axitinib. (b) OS from first-line therapy of patients treated with NIVO+IPI,stratified by TT regimens after NIVO+IPI, sunitinib or axitinib, IMDC all risks.”.

Figure 4 should read: “Figure 4. OS from first-line therapy of patients treated with nivolumab (NIVO) or nivolumab and ipilimumab combination therapy (NIVO+IPI). (a) OS from first-linetherapy of patients treated with NIVO, stratified by TT regimens after NIVO, sunitinib or axitinib. (b) OS from first-line therapy of patients treated with NIVO+IPI,stratified by TT regimens after NIVO+IPI, sunitinib or axitinib, IMDC all risks.” instead of: “Figure 4. PFS2 of nivolumab (NIVO) or nivolumab and ipilimumab combination therapy (NIVO+IPI). (a) PFS2 of NIVO, stratified by TT regimens after NIVO, sunitinib, or axitinib. (b) PFS2 of NIVO+IPI, stratified by TT regimens after NIVO+IPI, sunitinib, or axitinib, IMDC all risks.”.

These errors have now been corrected online.

