# Supplementary material for: Subgroup analysis of the AFTER I-O study: a retrospective study on the efficacy and safety of subsequent molecular targeted therapy after immune-oncology therapy in Japanese patients with metastatic renal cell carcinoma
Source: Jpn J Clin Oncol. 2021 Aug 4;51(11):1656–64. doi: 10.1093/jjco/hyab114 (PMC8558912; doi:10.1093/jjco/hyab114)
Supplement: Supplemetary_Tables_and_Figures_hyab114 [file supplemetary_tables_and_figures_hyab114.docx]

**Supplementary Materials**

Tables and Figures of “Subgroup Analysis of the AFTER I-O Study: A Retrospective Study on the Efficacy and Safety of Subsequent Molecular Targeted Therapy After Immune Checkpoint Therapy in Japanese Patients with Metastatic Renal Cell Carcinoma” Supplement

**Supplementary Tables:**

Table S1. Patient characteristics at the start of first targeted therapy after discontinuation of nivolumab and ipilimumab combination therapy, IMDC intermediate/poor risks

|  | | CheckMate 214(intermediate/poor risks) | | | | | |
| --- | --- | --- | --- | --- | --- | --- | --- |
|  |  | All  N = 16 | | Sunitinib  N = 6 | | Axitinib  N = 6 | |
| Sex, n (%) | Male | 14 | (88) | 6 | (100) | 4 | (67) |
|  | Female | 2 | (13) | 0 | (0) | 2 | (33) |
| Age, years | Median (range) | 70.0 | (46–82) | 60.0 | (46–73) | 77.0 | (70–82) |
| TTF of ICI, months | Median (range) | 7.9 | (0.0–27.6) | 5.5 | (0.0–27.6) | 5.5 | (1.4–12.6) |
| Reason for ICI discontinuation, n (%) | Progression | 10 | (63) | 4 | (67) | 3 | (50) |
|  | Adverse events | 6 | (38) | 2 | (33) | 3 | (50) |
| Surgery after ICI discontinuation, n (%) | Yes | 2 | (13) | 0 | (0) | 0 | (0) |
|  | No | 14 | (88) | 6 | (100) | 6 | (100) |
| ECOG PS, n (%) | 0 | 9 | (56) | 4 | (67) | 2 | (33) |
|  | 1 | 5 | (31) | 1 | (17) | 3 | (50) |
|  | >2 | 1 | (6) | 1 | (16.7) | 0 | (0) |
|  | Unknown | 1 | (6) | 0 | (0) | 1 | (17) |
| IMDC risk classification at 1st subsequent TT after ICI, n (%) | Favorable | 1 | (6) | 1 | (17) | 0 | (0) |
|  | Intermediate | 12 | (75) | 4 | (67) | 5 | (83) |
|  | Poor | 2 | (13) | 1 | (17) | 0 | (0) |
|  | Unknown | 1 | (6) | 0 | (0) | 1 | (17) |
| Primary tumor | Yes | 3 | (19) | 1 | (17) | 2 | (33) |
| Metastatic site | Lung | 9 | (56) | 4 | (67) | 3 | (50) |
|  | Bone | 6 | (38) | 2 | (33) | 1 | (17) |
|  | Brain | 1 | (6) | 1 | (17) | 0 | (0) |
|  | Liver | 3 | (19) | 0 | (0) | 2 | (33) |
|  | Lymph node | 6 | (38) | 3 | (50) | 2 | (33) |
| CRP ≥ upper limit of facility normal, n (%) | Yes | 11 | (69) | 4 | (67) | 4 | (67) |

Table S2. Overall response rate and BOR of targeted therapy after discontinuation of nivolumab and ipilimumab combination therapy (NIVO+IPI), subgroup in time-to-treatment failure of NIVO+IPI, reason for discontinuation of NIVO+IPI, targeted therapy regimens, IMDC intermediate/poor risks

|  | | All | | TTF of NIVO+IPI | | | | Reason for discontinuation of NIVO+IPI | | | | TT regimen | | | |
| --- | --- | --- | --- | --- | --- | --- | --- | --- | --- | --- | --- | --- | --- | --- | --- |
|  |  |  |  | ≥6 months | | <6 months | | PD | | AE | | Sunitinib | | Axitinib | |
|  |  | N = 16 | | N = 9 | | N = 7 | | N = 10 | | N = 6 | | N = 6 | | N = 6 | |
| ORR, n (%) | | 5 | (31) | 1 | (11) | 4 | (57) | 3 | (30) | 2 | (33) | 2 | (33) | 3 | (50) |
| DCR, n (%) | | 14 | (88) | 7 | (78) | 7 | (100) | 9 | (90) | 5 | (83) | 6 | (100) | 5 | (83) |
| BOR, n (%) | CR | 0 | (0) | 0 | (0) | 0 | (0) | 0 | (0) | 0 | (0) | 0 | (0) | 0 | (0) |
|  | PR | 5 | (31) | 1 | (11) | 4 | (57) | 3 | (30) | 2 | (33) | 2 | (33) | 3 | (50) |
|  | SD | 9 | (56) | 6 | (67) | 3 | (43) | 6 | (60) | 3 | (50) | 4 | (67) | 2 | (33) |
|  | PD | 2 | (13) | 2 | (22) | 0 | (0) | 1 | (10) | 1 | (17) | 0 | (0) | 1 | (17) |
|  | NE | 0 | (0) | 0 | (0) | 0 | (0) | 0 | (0) | 0 | (0) | 0 | (0) | 0 | (0) |

**Supplementary Figures:**


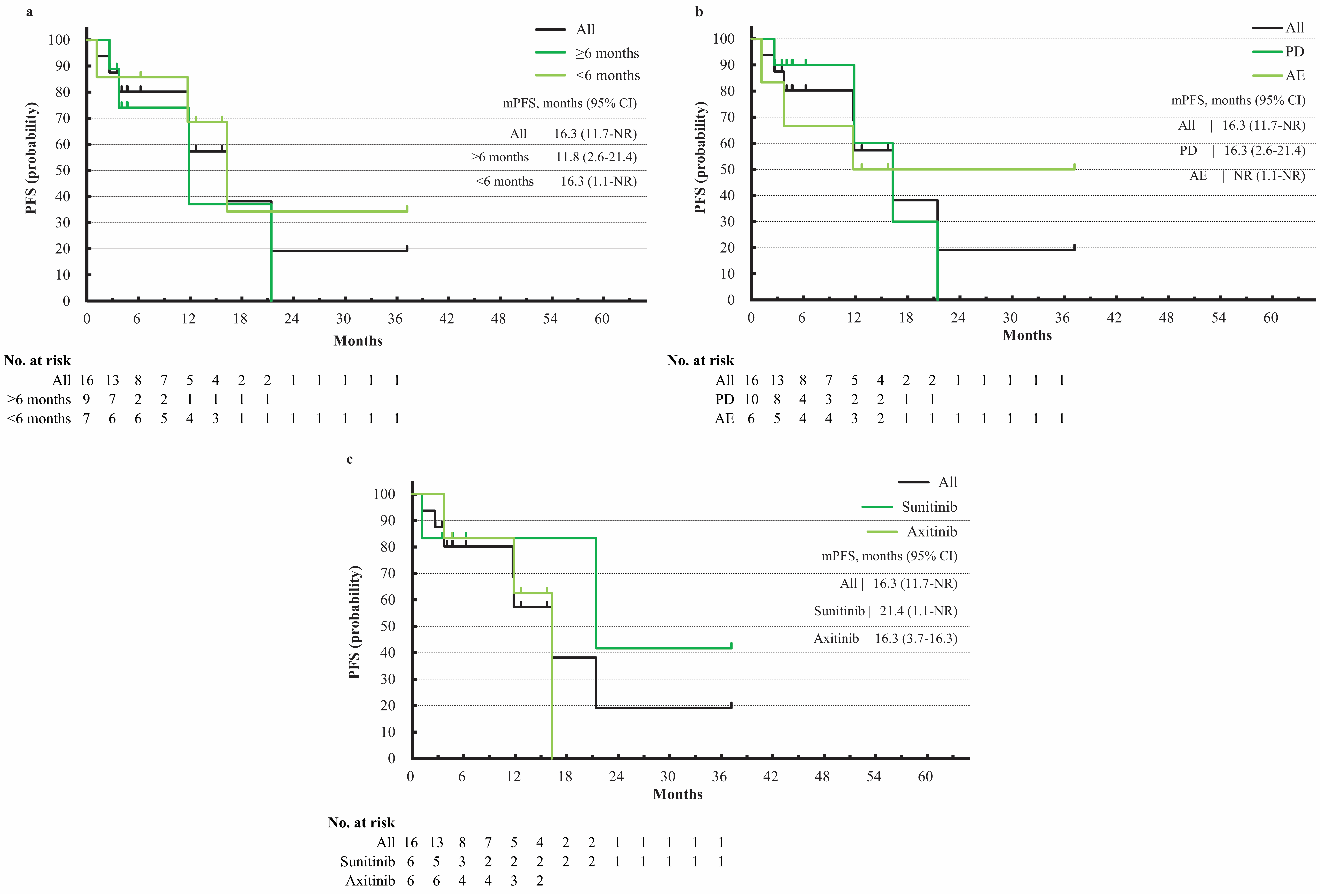


Figure S1. Progression-free survival (PFS) of targeted therapy after discontinuation of nivolumab (NIVO) or nivolumab and ipilimumab combination therapy (NIVO+IPI), IMDC intermediate/poor risks.

(a) PFS of targeted therapy after discontinuation of NIVO+IPI, stratified by time-to-treatment failure of NIVO+IPI, with a cutoff value at 6 months.

(b) PFS of targeted therapy after discontinuation of NIVO+IPI, stratified by reason for discontinuation of NIVO+IPI, disease progression or adverse events.

(c) PFS of targeted therapy after discontinuation of NIVO+IPI, stratified by targeted therapy regimens after NIVO+IPI, sunitinib or axitinib.


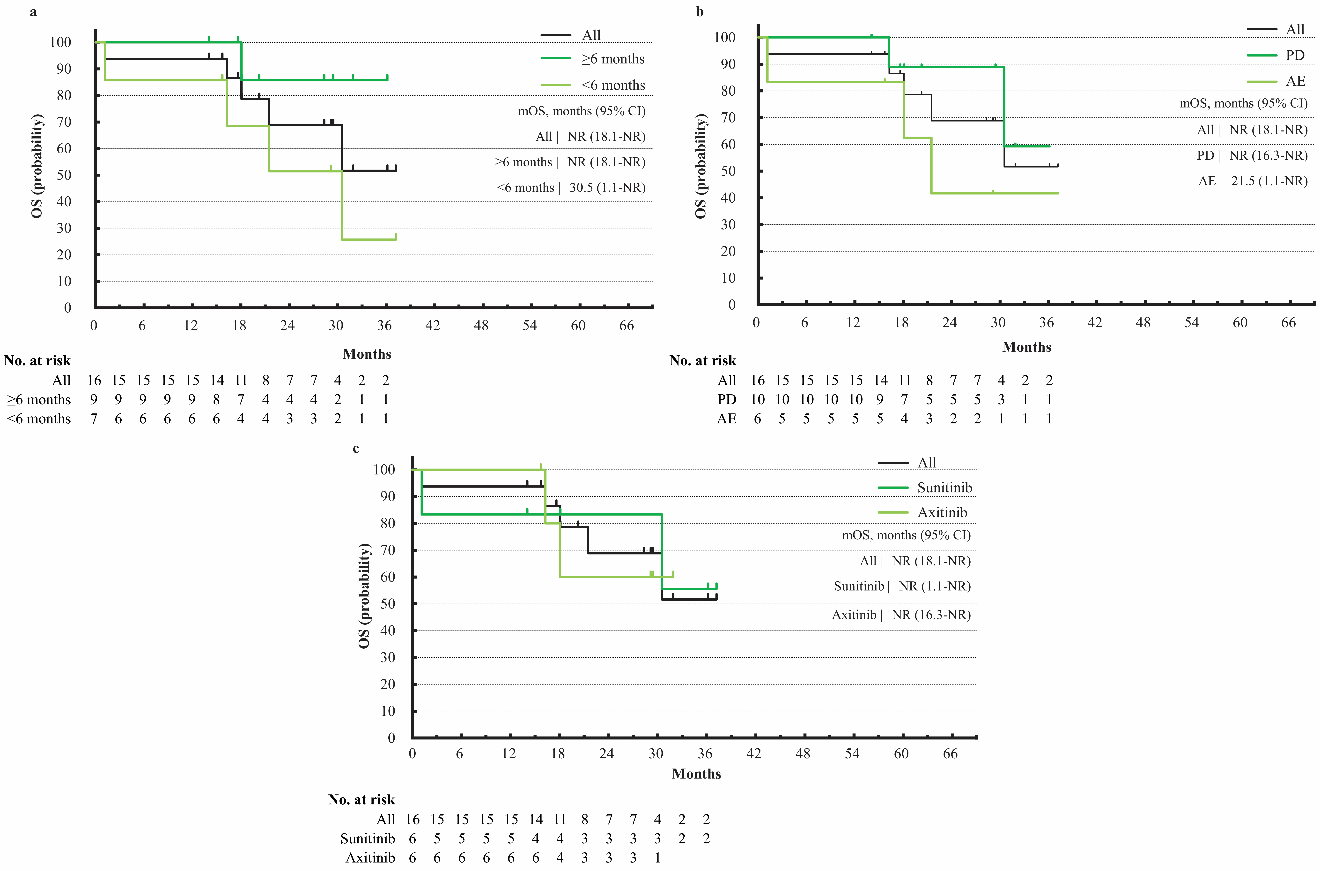


Figure S2. Overall survival (OS) of targeted therapy after discontinuation of nivolumab and ipilimumab combination therapy (NIVO+IPI), IMDC intermediate/poor risks.

(a) OS of targeted therapy after discontinuation of NIVO+IPI, stratified by time-to-treatment failure of NIVO+IPI, with a cutoff value at 6 months.

(b) OS of targeted therapy after discontinuation of NIVO+IPI, stratified by reason for discontinuation of NIVO+IPI, disease progression or adverse events.

(c) OS of targeted therapy after discontinuation of NIVO+IPI, stratified by targeted therapy regimens after NIVO+IPI, sunitinib or axitinib.


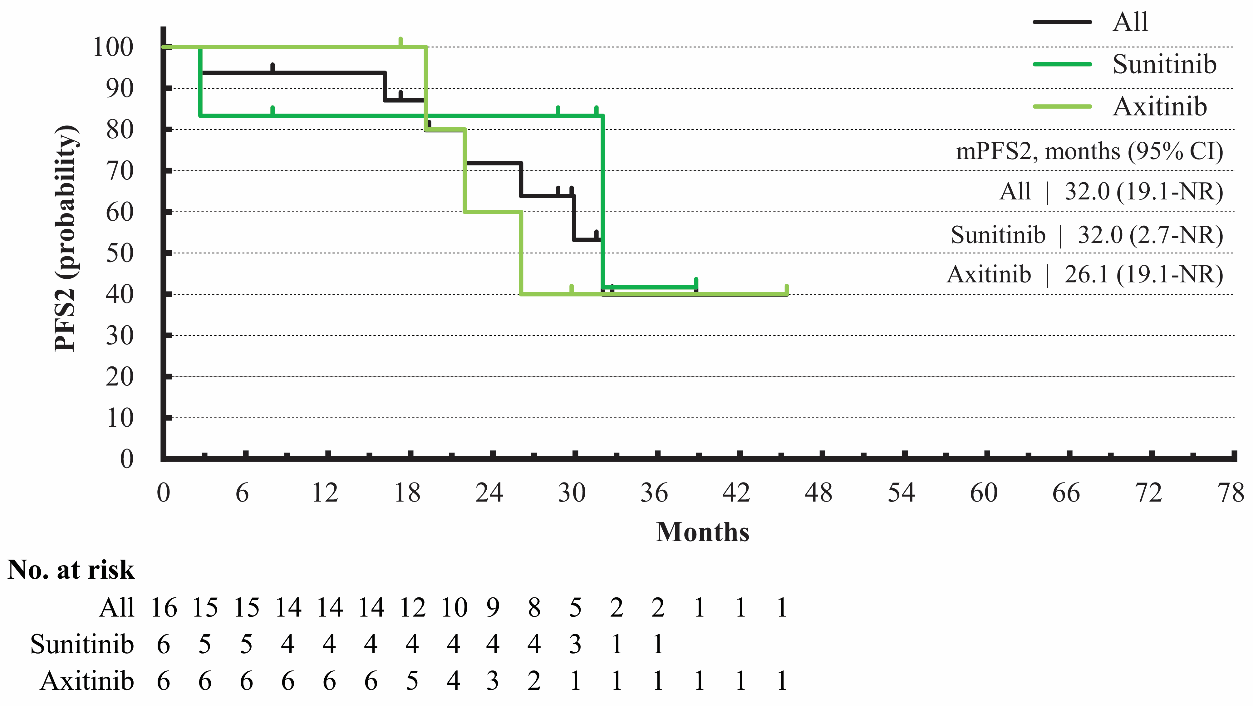


Figure S3. Progression-free survival 2 of nivolumab and ipilimumab combination therapy, stratified by targeted therapy regimens after nivolumab and ipilimumab combination therapy, sunitinib or axitinib, IMDC intermediate/poor risks


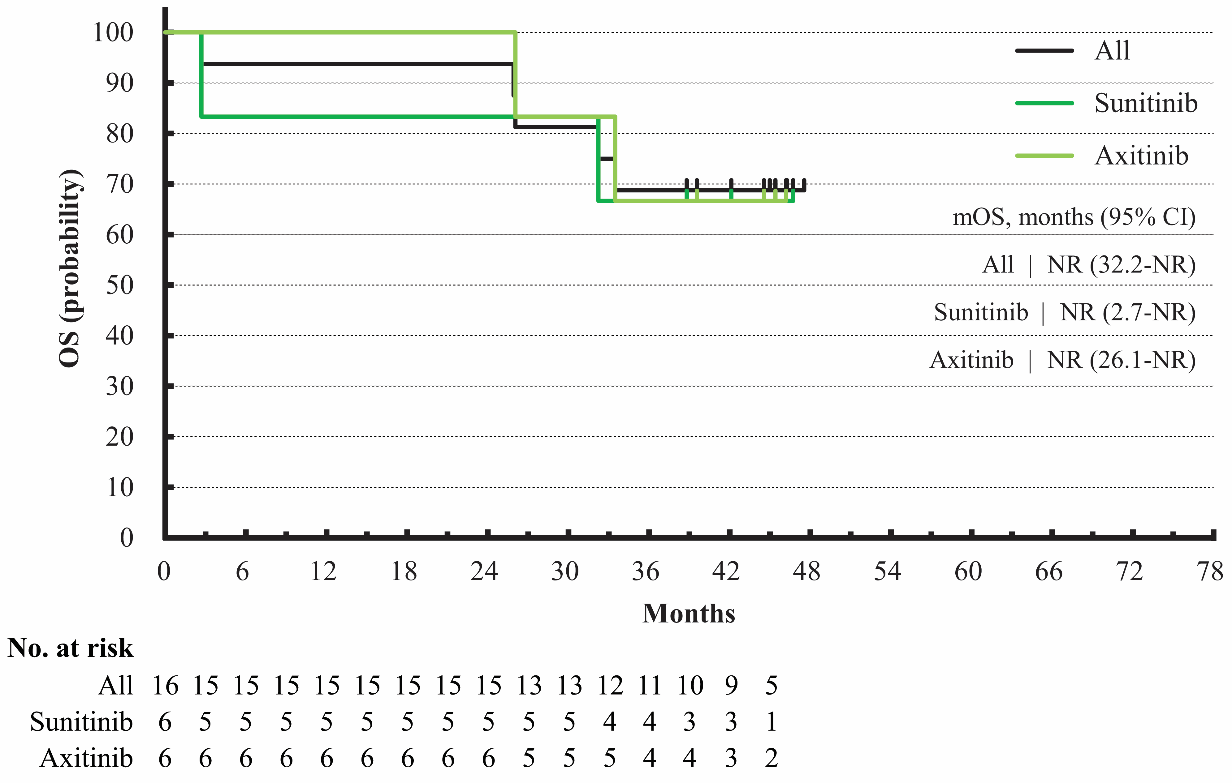


Figure S4. Overall survival form first-line therapy of patients treated with nivolumab and ipilimumab combination therapy, stratified by targeted therapy regimens after nivolumab and ipilimumab combination therapy, sunitinib or axitinib, IMDC intermediate/poor risks
